# Acceptability and satisfaction of a newly developed financial preparedness program for caregivers of older adults with a chronic illness

**DOI:** 10.1093/geront/gnaf148

**Published:** 2025-06-10

**Authors:** Katherine Judge, Lauren Stratton, Claire Grant, Monica Moreno, Sam Fazio

**Affiliations:** Department of Psychology, Cleveland State University, Cleveland, Ohio, United States; Center for Research and Education, Benjamin Rose Institute on Aging, Cleveland, Ohio, United States; Care and Support, Alzheimer’s Association, Chicago, Illinois, United States; Department of Psychology, Cleveland State University, Cleveland, Ohio, United States; Care and Support, Alzheimer’s Association, Chicago, Illinois, United States; Care and Support, Alzheimer’s Association, Chicago, Illinois, United States

**Keywords:** financial literacy, evidence-based programs, caregiving

## Abstract

**Background and Objectives:**

To address a gap in caregiver programming focused on financial literacy and preparedness, *Managing Money: A Caregiver’s Guide to Finances*, a 1-hour online program, was evaluated to determine program acceptability and participant satisfaction.

**Research Design and Methods:**

Caregivers of individuals with chronic health conditions were recruited to attend a one-hour program focused on (1) understanding the financial impact of caregiving; (2) providing information on legal and financial documents; (3) how to prepare for discussions about finances and financial planning; (4) lowering risks of financial abuse and fraud; and (5) creating a backup plan for changes in care needs. Results were collected longitudinally, following participants to ninety days after attending the program.

**Results:**

Overall, participants (n = 99) found the program highly acceptable, with the majority of caregivers agreeing or strongly agreeing the program: identified financial issues; provided important information; content was easy to read and understand; topics were clear and well-organized; activities were helpful; length was good; and caregivers would recommend the program. Program materials were rated helpful with up to 40% of participants reporting they used materials after attending the program.

**Discussion and Implications:**

Providing care for individuals with chronic health conditions poses significant challenges for informal caregivers. The impact of finances, knowledge base, and skills needed are often not included in an in-depth manner. The current program addresses this gap while also taking into consideration the need to develop pragmatic and scalable programs. The program may be useful in developing other targeted intervention protocols for caregivers.

Translational Significance Most caregivers experience financial difficulty of some sort in their role as caregiver; however, there is a significant gap in the availability of financial information in current programming targeting this population. This study found that a 1-hour online program focused on financial literacy and preparedness was acceptable and satisfactory to caregivers while also addressing specific needs. These findings suggest that the processes used in the development and evaluation of this program may be useful in addressing other gaps in programming with pragmatic interventions.

## Background and objectives

Providing care for a person living with chronic health conditions can be complicated and challenging due to the nature of the symptoms and changing roles and responsibilities. Approximately 53 million people provide care to an adult in the United States (AARP & National Alliance for Caregiving, 2020). Sixty-three percent of these caregivers are caring for an individual with a long-term chronic health condition (AARP & National Alliance for Caregiving, 2020) and more than eleven million of these caregivers are caring for a person living with Alzheimer’s disease or a related dementia (Alzheimer’s Association, 2024).

The financial impact on caregivers has been well documented. Specifically, caregivers often experience financial strain due to assisting in making financial decisions, cutting back on their own spending, and being unable to afford basic expenses (AARP & National Alliance for Caregiving, 2020; Alzheimer’s Association, 2024). The long-term financial impacts as a result of caregiving often include a stop in savings and an increase in debt (AARP & National Alliance for Caregiving, 2020). Over two-thirds of employed caregivers report that caregiving responsibilities affect their work including missing work, taking more time off, lower productivity, and/or leaving the workforce (AARP & National Alliance for Caregiving, 2020; Fakeye et al., 2022; Liu et al., 2020).

Greater financial stress and costs associated with caregiving have been shown to be related to caregiver distress and burden, symptoms of depression and anxiety, higher levels of role overload and role captivity, and decreased quality of life for caregivers (Adelman et al., 2014; Fan et al., 2023; Hong & Harrington, 2016; Lai, 2012; Liu et al., 2019; Liu et al., 2020; Nam, 2016; Vellone et al., 2007). Relatedly, caregivers with greater financial preparedness tend to have better physical and mental health outcomes (Adelman et al., 2014; Hong & Harrington 2016; Lai, 2012; Nam, 2016; Vellone et al., 2007).

Despite an identified want within the caregiving community for assistance in planning for their financial future, many caregivers do not have established financial plans for their future (DaDalt et al., 2016; Jolliff et al., 2024; Lauritzen et al., 2022). A survey commissioned by the Alzheimer’s Association revealed that only 53% of participants had made plans for their future care and financial needs and only 20% had discussed their care wishes with their spouse (National Alliance for Caregiving & Alzheimer’s Association, 2017). Without having conversations about finances, caregivers may not feel comfortable making decisions and may not have necessary knowledge about their loved one’s existing finances and benefits and can struggle accessing accounts to make financial decisions. More than 20% of caregivers of people living with chronic health conditions say it is difficult to find affordable services in their area resulting in paying out of pocket for expenses (AARP & National Alliance for Caregiving, 2020).

Relatedly, caregivers have expressed an interest in financial preparedness, including themes surrounding early care planning, understanding out of pocket spending, determining care values and preferences prior to needing care, understanding the financial situation of the person they are caring for, creating financial documents and advanced directives, and identifying financial and legal resources (DaDalt et al., 2016; Vellone et al., 2007). To address these areas, multiple studies have highlighted the importance of financial planning and preparedness particularly for dementia caregivers (DaDalt et al., 2016; National Alliance for Caregiving & Alzheimer’s Association, 2017; Zwingmann et al., 2020). However, there is a lack of evidence-informed and evidence-based educational financial programming for caregivers that directly address these issues within a single program (Sztramko et al., 2021). For example, financial issues primarily have been addressed within larger multi-component programs where the emphasis is focused on other educational and skill building areas and the financial component is minimal (Moore et al., 2020; Sztramko et al., 2021) and by providing additional financial resources and support by referring to local service organizations, such as Area Agencies on Aging and the Legal Aid Society (Judge et al., 2011; Shrestha et al., 2011).

As such there is a clear gap within the field for programs that solely focus on financial literacy and preparedness for caregivers. Providing financial literacy and preparedness education would be beneficial to caregivers in a variety of ways. First, understanding how to assess their own financial standing and how caregiving may affect it would help caregivers make more informed decisions and secondly, it would bolster caregivers’ self-efficacy and confidence in making financial decisions and executing plans.

To address this need for caregivers, *Managing Money: A Caregiver’s Guide to Finances* was developed and evaluated by the Alzheimer’s Association. An extensive environmental scan was completed to examine existing financial literacy and preparedness programs, services, and resources available and to identify gaps and opportunities for this current program. The environmental scan consisted of a comprehensive literature review and an evaluation of existing consumer ready programs. Second, a market analysis was completed to understand the financial needs, attitudes, barriers, and insights of caregivers to gain an understanding of what would be helpful for them to know. Lastly, focus groups were conducted with caregivers to validate the content and incorporate their thoughts and perspectives about the inclusion of interactive program activities. These findings were integrated into the content and delivery of the program.

## Research design and methods

### Program description

*Managing Money* was an educational program that helped caregivers learn about the costs of caregiving, benefits of early planning, and equipped them to undertake financial caregiving tasks on behalf of the person they are caring for. The program addressed seven key topics: (1) costs of caregiving, (2) how Alzheimer’s disease/dementia affects finances, (3) ­benefits of early planning, (4) conversations about finances, (5) avoiding financial abuse and fraud, (6) identifying financial and legal needs, and (7) creating a backup plan. Each of these topics was developed to build off of one another, allowing caregivers to become more familiar and comfortable with financial and legal topics throughout the course of the program. It was important to include specific information for dementia caregivers due to the on-going and progressive cognitive and functional declines they encounter and the resulting impact on their financial planning and preparedness. See Table 1 for additional details on the content included in each of these topics.

**Table 1.** Managing money topic content.

Topic name	Topic description	Interactive activities
Costs of Caregiving	The benefits of financial literacy, how caregiving may impact employment, the expense of formal care, and possible impact on caregiver physical and mental health	Check your knowledge questions Video from a caregiver
How Alzheimer’s Disease Affects Finances	The progressive nature of Alzheimer’s disease symptoms, the two-fold impact on finances including reduction in work and out of pocket expenses as care needs increase, and unique financial challenges associated with younger-onset Alzheimer’s, caregivers in the LGBTQ community, and lower income households	Check your knowledge questions Videos from a caregiver
Benefits of Early Planning	Who benefits from early financial planning and what those benefits include for the person with dementia and for the caregiver	Check your knowledge question Video from a caregiver Videos from a dementia care expert
Conversations About Finances	How to start difficult conversations surrounding finances and tools for success are provided for successful conversations	Video from a caregiver Worksheet: Having Conversations About Finances
Avoiding Financial Abuse & Fraud	Susceptibility to financial fraud and abuse, signs of possible problems with money or abuse to be aware of, and how to lower risk of abuse and fraud	Check your knowledge question Video from a caregiver Video from a dementia care expert Worksheet: Avoiding Financial Abuse and Fraud
Identifying Financial & Legal Needs	How to organize finances by taking inventory of assets and debts, identifying ongoing costs, and making future financial plans; financial resources and benefits, tips for legal planning, and how to find a financial or legal professional	Videos from a caregiver Worksheet: Covering Care Costs Worksheet: Organizing Legal Plans
Creating a Backup Plan	The aspects of a backup plan including planning for future care, taking care of the caregiver, and key resources for support	Video from a dementia care expert

An important aspect of the *Managing Money* program was the inclusion of interactive activities, which included “check your knowledge” questions, video clips, and printable activities. These activities were embedded throughout the various content areas of the program. The “check your knowledge” questions ensured caregivers understood the material and facilitated program engagement. Video clips also were integrated that highlighted real-life caregivers who shared their experiences of managing finances. Four printable worksheets were included for caregivers to use after the program and included: (1) Having Conversations About Finances; (2) Avoiding Financial Abuse and Fraud; (3) Covering Care Costs; and (4) Organizing Legal Plans. These interactive activities were designed to facilitate participant engagement, learning, and skill building.

The program was structured as a one-hour program delivered virtually through a videoconferencing platform and was offered to caregivers of persons with chronic health conditions (e.g., heart disease, diabetes, cancer, stroke, dementia). The Alzheimer’s Association is comprised of 77 local chapters throughout the United States which provide resources and support for caregivers and persons living with dementia. For this project, volunteer community educators received training on the program and were provided with a presenter’s guide containing content and delivery information on the program. Community educators were recruited from various local chapters and had previous experience delivering other programs at their chapters.

### Program evaluation

This study received approval from the Cleveland State University Institutional Review Board (IRB Study IRB-FY2021-159). Program acceptability and satisfaction data reported here were from the larger efficacy study that implemented a randomized controlled trial to examine the impact of the program across time. The Theory of Planned Behavior (TPB) was used as the overarching framework in guiding the larger RCT efficacy study along with the respective measures and hypotheses for examining efficacy (Ajzen, 1985; Ajzen, 1991). Specifically, the TPB was used for understanding, examining, and predicting behavioral intentions and completed behaviors in addition to knowledge and attitudes. The TPB suggests that an individual’s intention to complete an action influences the likelihood to follow through, particularly when there was a greater perceived sense of self-efficacy or control over the behavior. For the current paper, the focus was on the acceptability and satisfaction of the program. As such, the TPB was not used as a core component in guiding the methods, results, or discussion. Rather the goal was understanding key elements of the program thought to be linked to program acceptability and satisfaction along with discussing important facilitators of the program that address current barriers within the field in developing and implementing sustainable and pragmatic caregiver programs. Efficacy data examining the impact of the program from the RCT will be reported elsewhere.

### Measures

Data used to assess program acceptability and satisfaction consisted of single-item questions that were based on prior research (Judge et al., 2010). Evaluative items, if needed, were modified to reflect specific characteristics of the *Managing Money* program (e.g., activities liked, use and helpfulness of worksheets). Data were collected from participants after they completed the program (Time 2; T2) and at several follow-up time points including Time 3 (T3) 30 days after program completion; Time 4 (T4) 60 days after program completion; and Time 5 (T5) 90 days after program completion. All surveys were emailed to participants and participants who completed the program and all five surveys received a $125.00 gift card for their time.

Data collected at T2 consisted of a total of ten questions. Using a 5-point Likert scale, ranging from strongly disagree (1) to strongly agree (5), participants rated the extent to which they disagreed or agreed with eight statements about the program that included: identified financial challenges faced by caregivers; provided important information; content was easy to read and understand; topics were clear and well-organized; activities facilitated understanding of program content; the length of the program was good; whether they would recommend the program to other caregivers; and felt the program was important to other caregivers. Using a 10-point scale, caregivers also rated how likely they would be to recommend the program to their family, friends, or other caregivers. Lastly, caregivers were asked to select which program activity they liked the best. Data collected at T3, T4, and T5 asked caregivers whether they used any of the worksheets after attending the program (yes/no) and, using a 6-point Likert scale ranging from not at all (0) to extremely or very helpful (6), whether the worksheets were helpful.

### Analytic method

All data were entered, cleaned, and analyzed using SPSS (IBM SPSS Inc., Chicago, IL). Participant demographic data were analyzed using descriptive statistics including frequencies, means, and standard deviations. Each of the single evaluative items were analyzed using descriptive statistics including means, standard deviations, and frequencies. Independent t-tests and one-way analysis of variance (ANOVAs) were used to examine sample attrition.

## Results

### Participant characteristics

Six participant cohorts were recruited across a 6-month time period (from April 2021 through September 2021) from the following Alzheimer Association Chapters representing 6 different geographic regions: Illinois, Georgia, Greater Richmond (Virginia), Desert Southwest (Arizona), Houston Southeast (Texas), and Oregon Greater Southeast (Washington). Additional digital ads and social media postings through Association channels (e.g., ALZConnected) were utilized for recruitment. Study eligibility included individuals who self-identified as a family and/or friend informal (ie, not paid) caregiver who were providing care for an individual with a chronic health condition, such as Alzheimer’s disease, any type of dementia, cancer, diabetes, heart disease, multiple sclerosis, stroke, or another type of chronic health condition and were 18 years or older.

A total of 468 individuals initially expressed interest in the study. Of these, 322 participants enrolled in the study, completed the T1 baseline survey, and were randomly assigned to either the program condition (n = 164) or the wait-list condition (n = 158). Table 2 provides baseline demographic and caregiving characteristics for participants randomly assigned to the program condition (n = 164). Participants on average were 56.98 years old (SD = 15.29) and the majority were female (82.9%), Caucasian (73.2%), married (67.1%), and identified as heterosexual (89.6%). Most participants worked part- or full-time (56.7%), had a Bachelor’s or post/professional degree (74.4%), and had yearly incomes of $50,000 or more (54.9%). Participants primarily provided care to a parent/in-law (47.6%) or a spouse/partner (31.1%) and the length of caregiving was distributed across the following categories: less than 1 year (18.3%); 1 and 2 years (26.8%); 2 and 5 years (29.3%); and 5 or more years (25.6%). Most participants were caring for an individual with dementia (55.5%); individual with dementia and one or more other chronic health conditions (32.9%); and a smaller percentage of caregivers were caring for an individual with a non-dementia chronic health condition/s (11.6%).

**Table 2.** Demographic information T2 in program condition.

Characteristic	*n*	*%*	*M (SD)*
Caregiver type			
Dementia only	91	55.5	
Dementia and other chronic illness	54	32.9	
Chronic illness (not dementia)	19	11.6	
Age			56.98 (15.29)
Gender			
Male	25	15.2	
Female	136	82.9	
Other/missing	4	2.4	
Orientation			
Straight/heterosexual	147	89.6	
Bisexual	5	3.0	
Gay or lesbian	6	3.7	
Self-describe/prefer not to answer	6	3.6	
Race			
White	120	73.2	
Black/African-American	27	16.5	
Asian	5	3.0	
Hispanic	7	4.3	
Two or more/other race	2	1.2	
Prefer not to answer	4	2.4	
Income			
Less than $25,000	12	7.3	
$25,000–$49,999	36	22.0	
$50,000–$74,999	36	22.0	
$75,000–$99,999	25	15.2	
$100,000–$250,000	23	14.0	
$250,000 or more	6	3.7	
Prefer not to answer	24	14.6	
Level of education			
High school, GED, or equivalent	8	4.9	
Some college	34	20.7	
Bachelor’s degree	55	33.5	
Post/professional degree	67	40.9	
Marital status			
Married	110	67.1	
Single	47	28.7	
Widowed	1	0.6	
Other	6	3.7	
Work status			
Employed full time	75	45.7	
Employed part time	18	11.0	
Retired	44	26.8	
Unemployed	10	6.1	
Homemaker	5	5.5	
Other	12	7.3	
Relationship to individual with chronic condition			
Parent/in-law	78	47.6	
Spouse/partner	51	31.1	
Grandparent	10	6.1	
Other family relative	9	5.5	
Friend or neighbor	6	3.7	
Sibling	4	2.4	
Other	5	3.0	
How long caring			
Less than 1 year	30	18.3	
Between 1 and 2 years	44	26.8	
Between 2 and 5 years	48	29.3	
5 years or more	42	25.6	
Diagnosis type			
Alzheimer’s disease/other related dementia	139	84.8	
Diabetes	26	15.9	
Heart disease	21	12.8	
Parkinson’s disease	11	6.7	
Stroke or physical effects of a stroke	14	8.5	
Cancer	7	4.3	
Multiple sclerosis	2	1.2	
Other	35	21.3	

*Note.* N = 164; n = number of participants per group; % = percentage; M = mean; SD = standard deviation.

Diagnosis type will not add to 100% as participants were able to choose more than one option.

### Attrition analysis

Once enrolled in the study, the most significant attrition for participants in the program condition occurred between T1 and T2, with 61 individuals who did not attend the program nor complete the T2 survey and 4 individuals who did attend the program but did not complete the T2 survey. This resulted in a total of 65 individuals (or 39.6%) who dropped out of the study protocol between T1 and T2. Several analyses using independent t-tests and one-way analysis of variance (ANOVAs) were used to examine if there were any significant differences between participants who did and who did not complete the T2 survey. Demographic and caregiving characteristics examined included: age, marital status, work status, gender, orientation, income, relationship with the person receiving care, length of time providing care, education level, and impact of COVID-19 on their finances. A significant difference was found for the length of time providing care *F*(1, 162) = 4.18, *p =* .04), with caregivers who completed both the T1 and T2 surveys reporting they had been, on average, caregiving longer (M = 2.76; SD = 1.10) than caregivers who had not completed both the T1 and T2 surveys (M = 2.42; SD=.97). The scaling for this variable ranged from: 1 = less than 1 year; 2 = 1 and 2 years; 3 = 2 and 5 years; and 4 = 5 or more years.

### Acceptability and satisfaction

Acceptability and satisfaction data reported were from the participants (n = 99) who attended the 60-minute interactive education program and completed the T2 survey along with their respective data collected at T3, T4, and T5. Table 3 provides participants’ ratings after completing the program at T2. Mean ratings of the 5-point Likert scaled items ranged from 4.05 to 4.51, with a range of 82.8% to 91.9% of participants rating each statement as “agree” or “strongly agree.” The percentage of participants who “agreed” or “strongly agreed” with each statement about the program along with the mean and standard deviation included: *identified financial challenges faced by caregivers* (84.9%; M = 4.09, SD = 0.75); *provided important information* (82.8%; M = 4.05, SD = 0.67); *content was easy to read and understand* (91%; M = 4.49, SD = 0.68); *topics were clear and well-organized* (89.9%; M = 4.44, SD = 0.78); *activities helped in understanding of program content* (81.8%; M = 4.15, SD = 0.70); and *the length of the program was good* (86.8%; M = 4.23, SD = 0.71). Caregivers indicated they would *highly recommend the program to other caregivers* (91.9%; M = 4.50, SD = 0.68) and *felt the program was important to other caregivers* (82.9%; M = 4.51, SD = 0.86).

**Table 3.** Program acceptability ratings.

Variable	*T2 survey evaluation data*		
	*M*	*SD*	*% Rated agree or strongly agree*
This program identified the financial challenges I face when providing care for my loved one	4.09	0.75	84.9
This program gave me important information about how to manage money for myself and someone else	4.05	0.67	82.8
The program content was easy to read and understand	4.49	0.68	91.0
The topics were clear and well-organized	4.44	0.78	89.9
The activities in the program helped me understand the content	4.15	0.70	81.8
The length of the program was good	4.23	0.71	86.8
I recommend this program to other caregivers	4.50	0.68	91.9
How important is this type of education program for caregivers?	4.51	0.86	82.9
How likely would you be to recommend this program to your family, friends, or other caregivers?^a^	8.82	1.94	86.9

*Note.* N = 99; M = mean; SD = standard deviation; % = percentage.

a This item was rated on a scale ranging from 1–10, with 1 = very unlikely to 10 = very likely; percent rated 7 or higher reported.

Participants also evaluated which activities they liked the best throughout the entire program (Table 4) and indicated *topics covered in the program* (30.3%) and *videos of caregivers and Alzheimer’s experts* (24.2%) as the most highly endorsed activities. The activities of *developing your financial plan* (13.1%), *other—non-specified* (12.1%) and *exercises for saving money and reducing the risk of financial abuse and fraud* (10.1%) were all rated similarly. The *check your knowledge* questions were rated by 6.1% of the sample as the best.

**Table 4.** Activities liked most by participants—T2 survey evaluation data.

Response	Liked most	
	n	%
Topics covered in the program	30	30.3
Videos of caregivers and Alzheimer’s experts	24	24.2
Developing your financial plan	13	13.1
Other	12	12.1
Exercises to help save money, reduce the risk of financial abuse and fraud	10	10.1
Check Your Knowledge questions	6	6.1

*Note.* n = number of participants per group; % = percentage.

Another important aspect was examining whether participants engaged with program materials after completing the program. Participants were asked whether they used worksheets after attending the program at T3, T4, and T5. Worksheets included: *having conversations about finances; avoiding financial fraud; covering care costs;* and *organizing legal plans*. Table 5 indicated 40.4% of participants used a worksheet/s at T3; 38.2% of participants used a worksheet/s at T4; and 41.4% of participants used a worksheet/s at T5.

**Table 5.** Use of worksheets over the last 30 days.

Data collection time period	n	*% Yes*	n	*% No*	N	*% I don’t recall*
T3	38	40.4	43	45.7	10	10.6
T4	34	38.2	48	53.9	6	6.7
T5	37	41.4	43	47.8	8	8.9

*Note.* Time 3 (T3) N = 94; Time 4 (T4) N = 89; Time 5 (T5) N = 90.

n = number of participants per group; % = percentage.

Participants also were asked to rate how helpful worksheets were at T3, T4, and T5 (Table 6). For participants who did use the worksheets at T3, nearly a quarter or more of the sample rated each of the worksheets as “very” or “extremely helpful,” ranging from 24.4% to 30.8%. None of the worksheets were rated by more than 10% of participants as “not at all helpful” or only “slightly helpful.” At T4, for participants who did use the worksheets, nearly one-third or more of the sample rated each of the worksheets as “very” or “extremely helpful,” ranging from 28.1% to 36%. Only one of the worksheets, *covering care costs*, was rated by more than 10% as “not at all helpful” or only “slightly helpful.” At T5, of participants who did use the worksheets, nearly a quarter or more of the sample rated each of the worksheets as “very” or “extremely helpful,” ranging from 24.4% to 38.9%. None of the worksheets were rated by more than 10% of participants as “not at all helpful” or only “slightly helpful.”

**Table 6.** Helpfulness of worksheets

If you did use the following worksheet, how helpful was it?	*I did not use it (%)*	*Not at all helpful (%)*	*Slightly helpful (%)*	*Somewhat helpful (%)*	*Very helpful (%)*	*Extremely helpful (%)*	*Extremely or very helpful (%)*
Having conversations about finances							
T3	44.7	0	9.6	9.6	13.8	10.6	24.4
T4	38.2	2.2	6.7	10.1	16.9	12.4	29.3
T5	43.3	1.1	5.6	16.7	12.2	12.2	24.4
Avoiding financial fraud							
T3	41.5	1.1	4.3	11.7	22.3	8.5	30.8
T4	36.0	0	9.0	5.6	23.6	12.4	36.0
T5	41.1	0	3.3	11.1	23.3	12.2	35.5
Creating a monthly budget							
T3	46.8	2.1	3.2	12.8	13.8	11.7	25.5
T4	38.2	1.1	11.2	7.9	19.1	9.0	28.1
T5	38.9	0	4.4	8.9	22.2	16.7	38.9
Organizing legal plans							
T3	48.9	0	6.4	10.6	16.0	8.5	24.5
T4	38.2	0	6.7	11.2	20.2	11.2	31.4
T5	44.4	0	5.6	7.8	18.9	14.4	33.3

*Note.* Time 3 (T3) N = 94, Time 4 (T4) N = 89, and Time 5 N = 90.

## Discussion and conclusions

Though the financial burdens of caregiving have been well documented and the needs for financial preparedness have been identified by caregivers and researchers alike, there is an identified lack of available financially focused programming (Moore et al., 2020; Sztramko et al., 2021). The purpose of the current paper was to describe the newly developed program, *Managing Money: A Caregiver’s Guide to Finances*, along with presenting participants’ ratings of the acceptability and their satisfaction of the program. Assessing program acceptability and satisfaction was vital in determining whether the program was well-­received and appropriate for the intended population. Additionally, it was important to ensure the program was pragmatic and scalable by designing programs that fit within caregivers’ busy schedules and by providing the necessary educational content and skills.

Overall, the program received very high acceptability and satisfaction ratings as evidenced by participants agreeing or strongly agreeing with each of the 8 questions. Additionally, an overwhelming percentage of participants (91.9%) indicated they would recommend the program to other caregivers. This high level of endorsement was found to be in line with other caregiver programs that have been deemed highly acceptable (Gitlin et al., 2024; Judge et al., 2010; Qiu et al., 2019; Yous et al., 2022). Although varying in content and focus, these studies indicated the length, organization, topics, and/or program materials were acceptable and participants indicated programs were helpful and/or useful. Key features thought to underscore the success of the *Managing Money* program included the: (1) virtual delivery of the 60-minute program; (2) use of community-educators; (3) environmental scan, market analysis, and focus groups to identify key gaps and develop the appropriate evidence-informed content; and (4) interactive activities to facilitate participant knowledge, engagement, and action.

Close to 50% of caregivers do not have plans in place for future care or financial needs (National Alliance for Caregiving & Alzheimer’s Association, 2017). Based on the environmental scan, this was the first evidence-informed program developed that solely focused on enhancing caregivers’ financial knowledge base and skills. Previous programs typically embed financial aspects of caregiving within a larger multi-component program where the primary focus was developing skills for addressing non-financial aspects of caregiving (Moore et al., 2020; Sztramko et al., 2021). As such, these programs were not structured to provide the in-depth financial information, tools, and resources that caregivers need. *Managing Money* is an important program to meet the widespread need for programming that speaks to the nuances of the financial aspects of caregiving.

For many caregivers, attending in-person programs can be challenging due to time constraints and difficulties in finding care for the person they are providing care. Delivering the program virtually was a pragmatic and scalable approach. Offering a shorter program at one-hour in length also created an opportunity for easier attendance, again combating against potential time constraints and conflicting obligations. Additionally, the use of volunteer community educators represented a low-cost solution to implementing programming compared to Master’s level trained specialists. The use of community educators also helped to reach target audiences within their communities.

In terms of content, the program was informed by the results from the environmental scan and market analysis and further validated by caregiver focus groups. This systematic process ensured that the content addressed the gaps in the literature and provided caregivers with the necessary financial knowledge and skills. Given that caregivers were at different stages of their caregiving journey and had different levels of financial preparedness and resources, it was important that the interactive program tools (e.g., worksheets, video clips, check your knowledge questions) engaged participants and facilitated action when caregivers were ready. For example, the majority of caregivers who used the worksheets after completing the program indicated the tools were “*very*” or “*extremely helpful*.” These data highlighted that for some participants, worksheets were effectively being used up to 90 days after the program. Given the amount of information presented in the program, it was most likely that the worksheets enabled individuals to process, engage with, and then act upon setting and achieving financial related goals at their own pace.

It also was important to assess whether participants enjoyed the program activities especially for programs that are shorter in duration (e.g., 60–90 minutes) which typically have fewer activities and thus fewer opportunities for active engagement and learning. Overall, most participants selected the overall program content and the video excerpts as the best. Interestingly, the activity of developing a financial plan was not as highly endorsed. It may be this activity requires additional time outside of the program to fully engage and to be beneficial.

Although the current study found very strong evidence for program acceptability and satisfaction, there were several noteworthy limitations. While the program was offered virtually and was considered a more pragmatic and scalable approach, attending synchronous virtual programs may not be suitable for all caregivers. Alternative asynchronous programming should be explored as viable options for caregivers and whether participants find these types of programs acceptable and satisfactory. To address this, the Alzheimer’s Association developed and implemented an asynchronous version of the program for caregivers that is currently available and being utilized by caregivers. This program contains the same materials and content but is self-guided and self-paced.

Another limitation to consider was the use of technology which may be a barrier for some caregivers. Whether synchronous or asynchronous, these virtual programs require the use of a handheld device or computer and internet access. This is an important issue to consider when developing accessible programs for caregivers, especially those who may live in rural settings and/or do not have the economic means to attend virtual programs.

Although not addressed in the present study, another limitation stems from the lack of evaluating the program acceptability and feasibility from the vantage point of the community educators or the inclusion of qualitative data. This type of data would have provided additional evidence for the program including valuable insights into the delivery and potential areas for improvement.

Another study limitation stems from the sample of caregivers. Even though a comprehensive recruitment plan was implemented, including partnering with local organizations and targeted digital ads, the sample was rather homogeneous. For example, the majority of the sample was White, female, heterosexual, and college educated. Additionally, this study did not collect information about the caregiving context which may be useful in understanding the diverse financial needs of caregivers, such as whether caregivers live with the person they are caring for, or the number of hours spent providing care, and/or the characteristics of the caregiver’s support network. Future research should develop more extensive recruitment and retention plans that engage specific community leaders from under-represented groups in order to ensure the sample is representative.

Lastly, significant differences in the length of time providing care were found between caregivers who completed the protocol and caregivers who dropped out of the study. Specifically, caregivers were more likely to drop out of the study if they had been providing care for slightly less time. These caregivers may have had less time to participate due to their relatively newer caregiving role or they may not have perceived the need for this type of program yet.

Based on participant’s high acceptability and satisfaction ratings of the *Managing Money: A Caregiver’s Guide to Finances* program, there were several key learnings. First the program may serve as a model for developing other pragmatic and scalable educational protocols. A key tension in developing programs is balancing the “dose” or the amount of time the program requires participant engagement (ie, 1 hour versus 10 hours) with ensuring that the implemented program has high acceptability/satisfaction and efficacy. This tension may be most evident when trying to integrate larger dosed programs into existing social service and/or health care organizations as many organizations may not have the resources (e.g., staffing, training, reimbursement) to implement multi-session programs (Bass and Judge, 2010). As an example, when reviewing evidence-based programs for caregivers of people living with dementia listed in the Best Programs for Caregiving website (Benjamin Rose Institute on Aging and Family Caregiver ­Alliance, 2022), very few programs were implemented within a 1–2-hour format. Future research should focus on whether caregiver programs can be streamlined into smaller dosed programs while still maintaining quality.

Another key learning was from the successful development and implementation of the specialized program focused on financial literacy and preparedness. This was especially relevant given the lack of programs that directly address the financial aspects of caregiving. As such, there may be other specialized educational programs, such as communication and dementia-related behaviors, supporting independence, and person-centered care, that caregivers would find beneficial. Identifying these gaps requires a systematic approach in reviewing the literature (e.g., environmental scan, market analysis, focus groups) along with developing content that is easy to understand, useful, and well-organized.

Given the financial difficulties experienced by caregivers coupled with the lack of paid professional caregivers and consumer-ready resources (Wullert, Fuentes-Afflick, & National Academies of Sciences, Engineering, and Medicine 2024), there is a great need for financial preparedness programs that address these issues. Additionally, current policies across local, state, and federal initiative do not adequately address the needs of caregivers (Reyes et al., 2021) which inevitably negatively contribute and impact caregiver’s financial well-being. Overall, the program *Managing Money: A Caregiver’s Guide to Finances*, was rated as highly acceptable and satisfactory by participants. The interactive 60-minute synchronous program provided valuable educational information to caregivers of individuals with chronic health conditions, including Alzheimer’s disease and related dementias, and provided tools for facilitating future planning and action. In terms of next steps, the goal is to establish the program as an evidence-based financial educational program that addresses the needs of caregivers and encourages them to seek additional services, resources, and support. Furthermore, it is anticipated that both the synchronous and asynchronous versions of the program will be readily accessible to caregivers and utilized. Lastly, it would be of interest to examine the longer-term impact of the program in addressing financial related outcomes, such as caregiver self-efficacy, financial unmet needs and the related distress, and whether caregivers accomplished key financial related tasks.

## Data Availability

Study data, analytic methods, and methods are not available, and the study was not preregistered.
